# BAL-derived extracellular vesicles to test ex vivo for endothelial injury in HIV patients on antiretroviral therapy

**DOI:** 10.1093/ajrccm/aamag078

**Published:** 2026-03-16

**Authors:** Sarvesh Chelvanambi, Homer L Twigg, John Zagorski, Mark E Fraser, Nathan J Alves, Ling Chen, Navneet K Dhillon, Matthias Clauss

**Affiliations:** Pulmonary, Critical Care, Sleep & Occupational Medicine and Department of Emergency Medicine, IU School of Medicine, Indianapolis, IN, United States; Center for Interdisciplinary Cardiovascular Sciences, Division of Cardiovascular Medicine, Brigham and Women's Hospital, Harvard Medical School, Boston, MA, United States; Pulmonary, Critical Care, Sleep & Occupational Medicine and Department of Emergency Medicine, IU School of Medicine, Indianapolis, IN, United States; Pulmonary, Critical Care, Sleep & Occupational Medicine and Department of Emergency Medicine, IU School of Medicine, Indianapolis, IN, United States; Pulmonary, Critical Care, Sleep & Occupational Medicine and Department of Emergency Medicine, IU School of Medicine, Indianapolis, IN, United States; Pulmonary, Critical Care, Sleep & Occupational Medicine and Department of Emergency Medicine, IU School of Medicine, Indianapolis, IN, United States; Pulmonary & Critical Care Medicine, University of Kansas Medical Center, Kansas City, KS, United States; Pulmonary & Critical Care Medicine, University of Kansas Medical Center, Kansas City, KS, United States; Pulmonary, Critical Care, Sleep & Occupational Medicine and Department of Emergency Medicine, IU School of Medicine, Indianapolis, IN, United States

*To the Editor*

Although combined antiretroviral therapy (ART) effectively prevents the development of AIDS in HIV-positive individuals, the risk for comorbidities such as pulmonary diseases still prevails.^1^ Persistent HIV proteins both in lungs as well as circulating within extracellular vesicles (EVs) are candidates for causing comorbidities in HIV + patients on ART.^2-4^ HIV-Nef was shown to increase the release of EVs and to upregulate surface expression of the pro-inflammatory and proapoptotic EMAPII.^4^ Given the important role of EMAPII in pulmonary emphysema and viral acute lung injury,^4-6^ we asked whether BAL-derived EVs from a cohort of HIV patients on ART use EV-bound EMAPII to mediate proapoptotic activity. To underscore the translational relevance of our findings, we tested a neutralizing anti-EMAPII antibody in endothelial HIV-Nef-expressing transgenic mice, which have been previously shown to upregulate proapoptotic EMAPII in the vascular endothelium,^4^ as a therapeutic intervention by inhibiting endothelial cell death in vivo.

## Methods

We isolated EVs from acellular BAL fluid by sequential centrifugation and analyzed them as previously described.^4^ For EV-surface protein identification, isolated EVs were adsorbed to biotinylated antibodies immobilized on streptavidin-coated plates and stained with FITC-labeled anti-CD3 antibody. For ex vivo determination of endothelial apoptosis, caspase-3/7 activity was measured in human lung microvascular endothelial cells (HMVECs). Transgenic mice expressing Nef in endothelium using VE-Cadherin promoter and used for in vivo endothelial apoptosis characterization. For in vivo apoptosis detection, murine lungs were digested with collagenase Type-I, and stained with surface lineage markers (CD31, CD326, and CD45) and stress markers (EMAPII and cleaved caspase-3) and analyzed using flow cytometry. For statistical analysis, samples were deidentified, and differences between groups were assessed using one-way ANOVA followed by post hoc tests or Student’s *t*-test with Welch’s correction in Graphpad Prism v.10.

## Results

**Figure 1:**
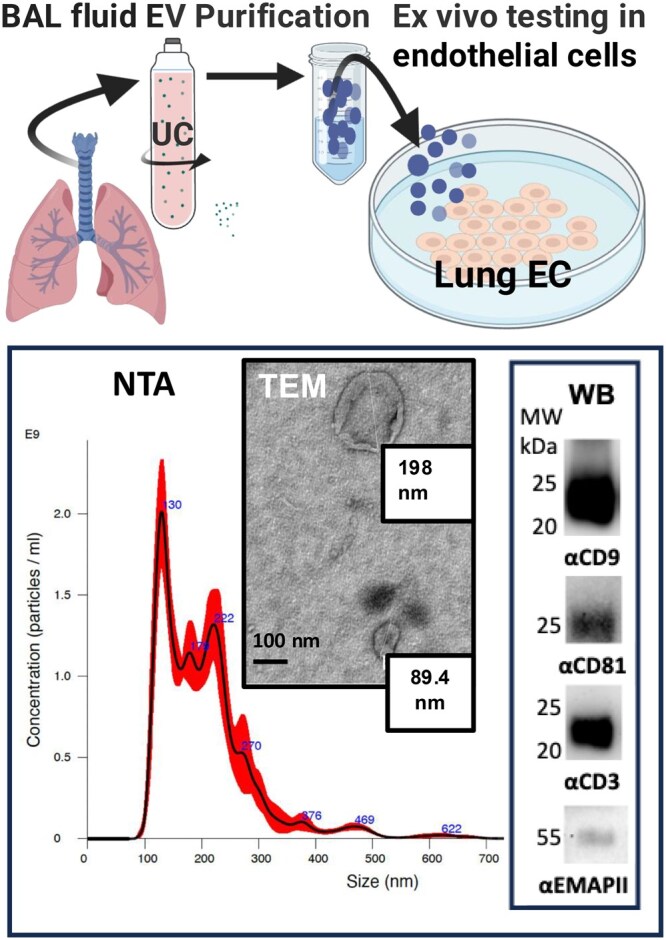
Schematic outline of purification of BAL-EVs and subsequent testing ex vivo (upper panel), which was created with BioRender.com. Isolated EVs demonstrated size distribution (NTA and TEM) as well as the presence of tetraspanin markers found on EV surface (lower panel).

**Figure 2:**
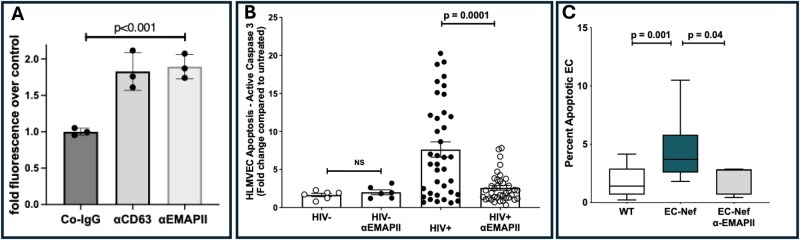
(A) BAL-EVs were adsorbed to biotinylated antibodies (CD63 and EMAPII) and then probed with fluorescent CD3 antibody. (B) BAL EVs from HIV + or HIV- patients were assessed by caspase 3/7 activity for apoptotic signaling. Shown mean± SD. (C) Percent of cleaved caspase 3 + endothelial cells (CD45-, CD31+) in endothelial -Nef tg mice vs WT littermates and after treatment with neutralizing EMAPII antibodies. (*n* = 5-9 mice). Groups were compared using one-way ANOVA with post-hoc tests (B and C) or Student’s *t*-test with Welch’s correction (A) using Graphpad Prism v.10.

BAL fluid-derived EVs were purified by sequential centrifugation and characterized according to *MISEV*^8^ using Nanoparticle Tracking Analysis (NTA), transmission electron microscopy (TEM), and Western blot (WB) analysis for EV (CD9 and CD81) and functional markers (CD3 and EMAPII) (Figure 1). Because EMAPII exerts its pro-apoptotic properties when it is present on the outside of cells,^9^ we captured EV-surface EMAPII using a biotinylated antibody and then detected with a fluorescently labelled anti-CD3 antibody (Figure 2A). Indeed, those EVs that adsorbed with anti-EMAPII antibodies, bound significantly more CD3 when compared to control IgG (Figure 2A). Of note, EMAPII adsorbed CD3 positive EVs at comparable levels to the well-characterized tetraspanin EV marker—CD63. To determine the proapoptotic activity of EV surface-located EMAPII in primary human lung endothelial cells, we tested EMAPII-neutralizing antibodies on BAL-EVs from a cohort of 37 HIV + patients on ART and 6 HIV- donors (Figure 2B). EVs from patients with HIV significantly upregulated apoptotic caspase-3/7 activation and EMAPII neutralizing mAb significantly reduced apoptosis.

To address the translational significance and potential therapeutic opportunity of targeting EMAPII, we used a previously described HIV relevant model of endothelial apoptosis in the lung. We injected EMAPII neutralizing antibodies into transgenic mice expressing the HIV viral protein Nef within the endothelium using a VE-Cadherin promoter. After two daily injections of EMAPII neutralizing antibodies, we found reduced cleaved caspase 3 in CD45-/CD31 + pulmonary endothelial cells (Figure 2C).

## Discussion

Our results suggest that the increased endothelial proapoptotic activity of EVs from HIV-infected subjects on ART is mediated by surface-presented EMAPII. First, using HIV-BAL derived EVs from three HIV patients on ART showed that biotinylated EMAPII-antibodies can recognize surface localization of EVs when compared to non-specific Isotype antibody capture (Figure 2A). Second, our HIV cohort-based ex vivo study (Figure 2B) provides further functional evidence that surface EV-bound EMAPII may link EVs to pro-inflammatory and pro-apoptotic vascular comorbidities including pulmonary disease.^4^^-^^10^ In addition, our results demonstrate that the combination of biotinylated EMAPII- and fluorescently labelled T cell antibodies can recognize surface localization of EVs from HIV-infected subjects on ART. The presence of the pan-T cell marker CD3 that EMAPII containing EV from BAL of HIV patients could represent a mode of communication amongst the milieu of cells within the lung. Furthermore, it may be hypothesized that surface-bound EV proteins, such as EMAPII, could contribute to the crosstalk between immune cells and endothelial cells, resulting in pulmonary vascular injury. However, additional studies are required to characterize their ability to not only induce endothelial apoptosis^11^^,^^12^ but also to act on other pulmonary cells, such as alveolar macrophages and epithelial cells. Even among endothelial cells, the effect of these EV on endothelial progenitor cells which help repair damaged endothelium needs to be studied based on our preliminary results using Nef-EVs.^13^^,^^14^ Furthermore, adoptive transfer studies in humanized mice could be employed to capture the contribution of these EV in vivo. Such studies could help determine the diverse effects of EV on pulmonary tissue. However, to determine the applicability of EMAPII neutralizing antibody to protect pulmonary endothelium, we employed a HIV relevant model with endothelial apoptosis. We used a transgenic mouse model expressing the HIV viral protein (Nef) in the endothelium using VE-Cadherin promoter.^4^ Our study demonstrates the in vivo ability of a therapeutic EMAPII-neutralizing antibody to significantly reduce endothelial apoptotic activity (Figure 2C). Similarly, the humanized version of this antibody demonstrated reduced parameters of cigarette smoke-induced lung emphysema, even if given after disease development.^15^ This suggests that neutralizing antibodies targeted at safeguarding pulmonary endothelial cells would form a new line of treatment for HIV patients with vascular disease.

## Supplementary Material

aamag078_Supplementary_Data
